# Transactions of the Medical Society of London

**Published:** 1817-07

**Authors:** 


### PART II.

### COMPREHENSIVE ANALYTICAL REVIEW OF MEDICAL LITERATURE.

"Tros, tyriusve, nobis nullo discrimine agetur."

### *Transactions of the Medical Society of London.*

( Concluded from vol. iii. p. 485. )

Art. 13. *Observations on the Use of the Bark of Swietenia Febrifuga.* By W. Roxburgh, M.D.

About the year 1791, Dr. R. published some observations on this bark; and between that period and 1806 (the date of the present paper) the medicine had been successfully used as a febrifuge in various parts of the world. Documents from numerous practitioners are here brought forward in support of Dr. Roxburgh's observations; but it is unnecessary to transcribe them, as they may be referred to in the volume itself.

Dr. R. has usually given the powdered bark in substance, mixed with cold water, in doses of from 20 to 60 grains, generally the latter dose. The first day it usually proves considerably laxative, but less so afterwards. He could observe no other sensible mode of its operation, except the entire removal of the disease, and an improved appetite. Little or no nausea, or any kind of uneasiness, attended its use. In some gangrenous and other bad ulcers, the Doctor continued this medicine for five or six weeks, three or four times a day, without his observing any visceral obstructions to ensue. Affections of the spleen [ague cakes], so common after the use of Peruvian bark in hot climates, were not observed to follow the exhibition of the Swietenia.

Art. 14. *Case of Hydrophobia.* By —— White, Esq. Assistant Surgeon of the Westminster Infirmary.

This was a young man 19 years of age, who was seized with symptoms of Hydrophobia six weeks after the infliction of the bite. The phænomena were similar to those recorded in other histories of this dreadful malady, and the result was the same — death. Blood-letting, opium, digitalis, were the remedies employed, but without effect.

"The only fact (says Mr. White) we have gained from the treatment of this case is, that the sensorial powers cannot be affected by the administering of opiates, which class of medicines may, in future, be laid on the shelf. Indeed, the presence of hydrophobia appears to render the body not amenable to the operation of medicine in any degree." 188.

Art. 15. *Miscellaneous Remarks on Medical Subjects.* By James Sims, M. D.

After sixty years' study and practice of physic, Dr. Sims has retired, and conceives that he cannot be better employed in conquering the indolence of age, than in collecting such observations as he has made in his own long and extensive practice.

"I cannot help wishing (says he) that the press gave us more *empirical* observations from aged physicians, than fine-drawn theories and hypotheses by young ones."

To this we say, Amen! But why call them *empirical* observations? Yet the old gentleman does not disdain to rear up a delicate little *theoretical* chicken as a bonne bouche for himself and the Society. It comes forth, too, in the plumage of an *empirical* observation! It is this: Boerhaave having said (or somebody for him), that "he should be able to cure almost every disorder incident to the human body, if he could produce, *at pleasure*, an ague," Dr. Sims proposes transporting the inhabitants of Bedlam, and all other lunatic asyla, to Essex and Lincolnshire — for the benefit of the air!

Blood-letting Dr. S. has not found serviceable in mania; but in melancholia the case is often different.

"If the patient complains much of oppression on the head or brain, and seems much paler and weaker than preceding circumstances would lead us to expect, I have often seen great service from taking blood either by the lancet, by cupping, or by leeches applied to the temples. In this latter case I have also found wine, spirits, and all cordials and stimulants, eminently hurtful, though they would seem so proper to raise the pulse and spirits. This elevation is most certainly procured by bleeding, when that has been properly administered." 192.

Dr. S. thinks that the alarming increase of insanity in this country is owing to the following causes: 1, The too great use of animal food and high sauces. 2. The inordinate consumption of strong vinous liquors: spirits, he thinks, produce phrenzy and sudden death. 3. The *auri sacra fames,* or rage for money.

2. *Second Empirical, or rather Empirico-hypothetical Observation.*—The hot-wells of Bristol, some years back, deservedly acquired a character for curing many bad cases of consumption. At present their estimation is on the wane, owing, as Dr. S. thinks, to the imprudence of the builders, and not to the ultra-approbation of Beddoes, as some of the proprietors imagine. The original low, warm, sheltered situation, where the patients resided, is now changed to a bleak hill nearly three hundred feet in perpendicular height above the old Wells. This is a very inconvenient site for pulmonic patients, as it is almost constantly visited by violent gusts of north-west winds from the Welsh mountains, and from the sea up a deep valley gradually more and more pent in, until they are at last discharged, with their accumulated force, upon Clifton. Most of our consumptive patients are seized with the complaint in hilly situations; and therefore a low ground would probably be favourable to recovery. The following case strongly corroborates the theory of Dr. Wells:

"A young lady, obviously far gone in a pulmonary consumption, applied for my advice since I quitted practising my profession for gain. As I thought she could only be saved by uncommon methods, I advised a removal to a very aguish part of Essex. As I thought she might require medicines whilst there, I agreed to accompany her to a relative's house on the spot. The consequence was, that, within three days, she was seized with a tertian intermittent, and did not cough once after the second fit of the ague. I kept her there until she had seven or eight regular returns of the paroxysm, and then, bringing her to London, easily stopped the ague with proper remedies." 198.

Much do we fear that few such recoveries from *genuine phthisis* would follow a Lincolnshire or Essex ague. The case, however, is worthy of record; and the practice of imitation in such an opprobrium medicorum.

3. *The Brine Bath.*—Dr. Sims has been above forty years in the habit of ordering, for various patients, a brine bath, made in the following manner:

"Take as many gallons of water as will fill a third of the bathing-tub you intend to use; to this add about as much common sea salt as there was water. If the water be boiling at the time of using it, the whole will be immediately dissolved; if not, some of the salt will remain granulated in the bottom at first, but which will be gradually dissolved."

This bath will keep good for years. Any scum that gathers on the top is to be carefully taken off, and all dust to be kept out. Nervous weak persons, adds our author, often cannot bear a cold bath of common water, but will bear this with ease. People come out of it with a glow on their skin, and very agreeable sensations.

4. Infallible *Styptic for Epistaxis.*—This is made by lighting a piece of common cork at a candle, and, as it burns, by scraping off gently the black burnt powder. It is to be strongly and repeatedly snuffed up the bleeding nostril, and—"I never knew it to fail"—except in one instance. "Indeed its effect, in these cases, is so instantaneous as to seem like magic." p. 202. This is a genuine specimen of *empirical* observation.

5. *Sulphate of Zinc.*—Dr. S. lauds this emetic in high terms.

"Other emetics (says he) produce their effect by bringing the diaphragm and abdominal muscles into very strong contraction, so as to squeeze out the contents of the stomach by that contraction. But the white vitriol *appears to evacuate simply by the contraction of the stomach itself*, without any assistance from these muscles." 204.

Has Dr. S. made experiments similar to those of Magendie on this subject? We fear not; and we hesitate not to affirm, that this is an *empirical* observation founded on an erroneous *hypothesis*. Dr. S. exhibits this emetic in doses of one to two scruples.

6. Carrot poultice in cancerous and canceroid ulcerations. This is now too old to be noticed here.

7. *Deafness.*—Dr. Sims has long been subject to occasional deafness in one or both ears, which he has found to be relieved better by the following than any other plan. He begins by dropping every night into the affected ear a little strong solution of soap in water, lying down upon the opposite side until he falls asleep. Having done this for several successive nights, he syringes, in the mornings, the ear with a gently warm solution of soap and water, to which a little brandy is added.

8. *An infallible Cure for the Tooth-ache.*—Dr. S. was long afflicted with this disagreeable companion; but cured himself by resolving to put nothing in his mouth hotter than his own blood. Dr. S. was led to this cure by a curious piece of philosophy; namely, because "he knew that hard bodies (for instance the teeth) were most liable to be dilated and contracted by heat and cold." At this rate *real silver* would make a better thermometer than *quicksilver!*

9. *Cramps in the Limbs of gouty People.*—Dr. Sims, conceiving that these arose from *too free* a return of blood from the lower extremities, when the person was in bed, and from the want of garters, as in the day. Garters, therefore, seemed the cure; and the first fee the Doctor ever received was for prescribing a pair of garters.

10. This article is a desperate long one on the Materia Medica; but as we were unable to comprehend its drift, we refer the reader to the work itself. Another long article, on the Language cf the Fingers, Elbows, &c. follows; but as it is not calculated for our class of readers, we shall pass it over.

ART. 16. *Impregnation, when the Vagina was scarcely permeable.* By JOHN SQUIRE, M. D.

MRS. K. a young woman, fell in labour, and was attended by a midwife, who called in Dr. Squire. On examination, no entrance could be perceived into the vagina. On more minutely examining the parts, a small aperture was observed in the anterior part, near the orifice of the urethra, just large enough to admit the introduction of a small goose quill. Through this the end of a director was pointed towards the perinæum, and the intervening substance was divided along the groove of the director with a scalpel. The hæmorrhage was trifling, labour came on, and all did well.

ART. 17. *Extraordinary Tumour, supposed Aneurismal.* By JOHN MOODIE, M. D.

H. E. of a strong and otherwise healthy constitution, was brought to Dr. Moodie's house, July 3, 1803, and gave the following history of his case:—In the preceding March he sprained or hurt his left arm, which was followed by a painful and acute sensation in the shoulder of the same side. This partly went off in about a fortnight, but a slight enlargement of the shoulder joint was suspected, and a blister was applied, which gave some relief. Pain, however, continuing about the articulation, a dislocation was suspected, and the usual means of reduction put in force without success. The tumour increased rapidly after this; and when Dr. M. saw it, it was the size of a person's head. It was tense, somewhat discoloured; prominent at the superior part, where the skin is very thin. There is great uneasiness on pressure, but no perceptible pulsation. Being punctured at one point, a tea cupful of blood was discharged, and the orifice closed. At the depending part, the veins were varicose and reticulated. Accompanying this affection, were various symptoms of constitutional commotion and irritation. The pulse at the wrist of the arm affected was small, intermitting, and hardly perceptible; at the other, quick and hard. In a consultation, it was considered *aneurism.* The tumour rapidly increased, till it was three feet ten inches in girth. Small quantities of blood frequently issued from the surface of the tumour; and about a week before the patient's death, a large wash-hand basin full was discharged at one time. He sunk on the 20th of August.

Now we believe that this Journal will by this time have exhibited a parallel case, under the denomination "*Hæmato-scrofulous Tumour,*" where amputation saved life, and dissection of the limb showed that aneurism had nothing to do in the business. We are convinced that the above case would, on dissection, have shewn precisely similar appearances.

Art. 18. *Case of Hydrophobia, with the Appearances on Dissection, &c.* By Thomas Walshman, M. D.

A boy, in forcing a bolus down a dog's throat, the dog being ill but not suspected of madness, was slightly wounded by the dog's teeth. No notice, however, was taken of the accident, and the wound healed. About four months afterwards hydrophobia completely developed itself. Blood-letting and opiates had no effect in arresting the fatal progress. There was no other morbid appearance, except that

—"the upper orifice, or cardia (of the stomach), was a little more vascular than usual, but not inflamed. In examining various dogs that died of hydrophobia, there was no other morbid phænomenon to be seen; the rugæ of the stomach being numerous and prominent."

That this dreadful poison cannot be communicated from one human being to another, may be inferred from the following history:

"A gentleman, far advanced in years, was afflicted with hydrophobia, and while he was in that state, desired to salute his daughter: he seized her cheek with so much violence between his teeth, that we had much difficulty in disengaging her from him; and she did not wash off the saliva deposited in the wound." She did not suffer from the circumstance. p. 251.

Of the prophylaxis, Dr. W. thinks there is nothing but excision that can be depended upon.

"I have had several cases of persons bitten by dogs afflicted with hydrophobia, in which the part has been excised; not one of them has been afflicted with this disease, and it is now many years since the operation was performed on some of them." P. 255.

Dr. W. recommends a trial of extract. hyoscyami in as large doses as the patient can bear; this remedy never yet having been tried.

### Art. 18.—*Cases of Gout, cured by Elaterium.* By J. Green, Esq.

A. C. Esq. a hearty man, turned of 60, complained of extreme pain in the foot and ankle, which were so much inflamed that he could not bear the slightest touch. The preceding night had been passed in torment. Pulse full, about 96.

℞. Inf. sennæ ℥iss; tinct. sennæ syr. rosæ ana ʒj; elaterii $\frac{1}{3}$ partem unius grani; tinct. opii, gt. x; tinct. digitalis, gt. iv. M. f. h. vesp. sum.

He continued restless and in much pain until three in the morning, when he felt nausea, and had a very copious alvine evacuation; three more stools followed, after which he enjoyed three hours sound sleep. He then repeated the draught, and by ten o'clock was able to walk down stairs with little uneasiness. The second draught made him sick, and procured several loose motions. On the third day he felt quite well and walked out. About a week after, a walk in a sharp frost brought on another severe attack of the gout. I saw him two days after, incapable of moving, and in excruciating pain, when he earnestly solicited the same medicine he had tried before. The first draught brought up a great deal of bile, and procured him several loose motions. It was repeated at bed-time, with the same effects; the first part of the night being rendered uncomfortable by frequent scalding stools. At five o'clock he had four hours refreshing sleep; and on waking he took a third draught, which kept up the alvine discharge. Between eleven and twelve, I found him walking about, the pain and gout having left his knee. He took no more medicine, and remains quite well. Mr. Green has not found any evil attending the antiphlogistic plan, convinced, from experience, that heating medicines and flannel wrappers are injurious. Where there is much inflammation, Mr. G. applies leeches. The following case, as well as the former, shews the good effects of purging in such cases:—Mr. S. about 60, accustomed to spring attacks of the gout, which last three or four months, was seized with gout in both feet, about the middle of January. On the second day of his illness he took, by my advice, three of the foregoing draughts at proper intervals, which vomited and purged him pretty smartly; at the end of two days he was down stairs, having exchanged the three months confinement he dreaded, for trifling debility, which he soon expected to get over. He is now taking draughts occasionally of equal parts of inf. gent. c. et inf. sennæ, with a view of strengthening the stomach and promoting the discharge of colluvies from the bowels.

ART. 19. *Cases of fatal Obstruction in the Bowels, with the Appearances on Dissection.* By G. DAMANT, Esq.

JOHN MORRIS, ætat. 50, had been afflicted for twenty years with temporary bowel obstructions, attended frequently with stercoraceous vomitings, which continued about twenty-four hours, when the patient was relieved by natural evacuations. The vomiting had continued four days, when, on calling in medical aid, the abdomen was found greatly distended; the pain not more than might be expected from such distention. From twelve to fourteen pints per diem of fæculent matter was vomited up; not more than a pint and a half of aliment was taken. His thirst was moderate, and his pulse not very quick. The physician who was consulted pronounced him in imminent danger. He directed a simple enema, and a grain of calomel, with five grains of jalap every hour till the next day. The vomiting continued, and no effect was produced, nor would the enema remain in the rectum. Mr. Damant endeavoured, in vain, to pass a large bougie. Sumat. sulph. magnes. ʒj, 2da quâque horâ. After throwing up two or three tobacco enemas, the bougie passed a little higher. The smoke of tobacco was tried nearly an hour, without inducing syncope. Sum. olei ricini coch. i. min. omni horâ. Nine ounces of the oil were taken, and the patient expired on the 11th day of the disease.

*Sectio cadaveris.* The small intestines unusually distended, bearing strong marks of inflammation. The valvula coli perfect; no obstruction presented itself until we examined the colon; it was contracted from its beginning to the termination of the rectum, and filled with a tenacious whitish substance which scarcely admitted the finger to pass. Water would not find a passage.

A. B. ætat. 60, had been in the habit of taking purgatives for extreme costiveness. From some cause, she remained a week without evacuations, when, on the failure of her usual remedies, she applied to her medical attendant. Finding considerable pain and sickness, he took from her eight ounces of blood, and directed her to take some opening medicine. I saw her the next day, and directed a larger quantity of blood to be taken. This did not appear so much inflamed as the last. Various remedies were tried; much difficulty in throwing up an enema. Some liquid fæces were at last voided, but the sickness increased, and the patient vomited only fæculent matter. The tension of the abdomen was considerable, and death took place on the fourth day.

*Sectio cadaveris.* The abdomen exceedingly enlarged, in appearance like a steatomatous tumour. The adipose substance extremely hard and thick; when the peritonæum was punctured, a quantity of very offensive air rushed out. The whole intestines highly inflamed, the jejunum almost in a state of gangrene. The large and small intestines distended with air and fæces; the colon greatly enlarged and full of fæces. Four inches from the anus there was a stricture in the rectum, which even excluded fluids. The liver was small, and had the right lobe adhering to the peritonæum. The other viscera were sound. The author of this paper observes, that these diseases are more common than is suspected; and if the stricture or adhesion is high up, we must despair of removing it. False delicacy frequently induces a state of costiveness, which, by the lodgment of fæculent matter, may cause a permanent contraction, either by the effusion of coagulated lymph, or by mere irregularity in the peristaltic motion.—The following case seems to justify such an opinion:—General E. returned from the West Indies with so considerable a number of fellow passengers, as to render the cabin accommodations very irksome to a delicate man, and for several days the General found no convenient opportunity of relieving himself. He subsequently suffered from long costiveness, and for a considerable time had no evacuations but by the œsophagus. He at length was relieved, but only by liquid stools; and, after a return of the complaint, died. In the post mortem examination, a stricture was discovered high in the intestines.

### Art. 20. *Effects of Diet in Stomach Disorders.* By J. P. Dale, Esq.

The following cases are not new, but the success which attended the mode of treatment would seem to shew that much may be done by mere attention to diet.

*Case* 1. Mrs. W. ætatis 36, had laboured under a severe complaint at her stomach for many years; for which she had been variously treated, with temporary relief. She said that in her 18th year, while walking, she suddenly felt as if something heavy had fallen into the stomach, attended with severe pain. The family surgeon failed in procuring relief, and the physician who was consulted, after having tried various remedies unsuccessfully, declared the complaint to be rheumatic, and advised her to bathe and drink the Buxton waters. After having continued this course about a month, she returned home much better. She had, however, once in six weeks, a return of the pain in her stomach; which lasted, with interruption, from two weeks to four or five: she occasionally used the cold bath, which seemed to give some relief. This distressing complaint continued until about a year after her marriage, when she became pregnant, and was free from these attacks for two years. At this time, anxiety brought on a violent fit of the old complaint; the pain always increased after eating, with flatulence, acidity in the stomach, bilious vomiting, and in short every symptom of complete dyspepsia. Mr. Dale advised her to take such food only as with difficulty passes into the acetous fermentation; animal food, with a small quantity of hard biscuit and water. The day after she was put on this regimen, she had received great relief, and became every hour better, so that at the end of three days, she said she felt herself better than she had been for years. She continued this diet, without any vegetables, for fifteen days, occasionally taking a little hydrosulphuret of ammonia, and an aloetic pill with ammonia ppt. to keep the bowels open. Sickness and a lax state of the bowels compelled her then to desist from that diet, and live on milk only, which soon removed those complaints. The stomach has now recovered its healthy action, and she continues perfectly well, except when she has neglected her precautions.

*Case* 2. Mrs. B. ætatis 39, a married lady, with four children, of a spare habit, active and temperate, and in general healthy, was seized with a very sharp pain at the neck of the bladder and across the loins. Urine scanty, but frequently passed, containing no sand or gravel, but high coloured. She had tried various medicines, particularly the alkaline mephitic water, some time, with little or no effect; and despairing of permanent relief, had for nine years ceased to ask advice. She seemed listless and irritable; her eyes were red, and peculiarly wild; she never had felt any sickness at the stomach. Injiciatur enema emolliens statim, post catharsin sumat haustum anodynum. Her food was desired to be light; her drink as much milk as she pleased, mixed with two parts of barley water. Two days after, Mr. D. saw her, and found her urine of the colour of strong coffee; the odour highly pungent, and resembling caustic volatile alkali. Such she said had been the case during her whole illness. Mr. D. finding her diet consisted of almost entirely animal food, advised her not to eat any. She abstained from it, as much as possible, and grew rapidly better; but, on returning to her old diet, suffered a relapse. She therefore returned to her vegetable diet, and in a few months her health became perfectly good, her urine having gradually become natural, from the abstraction of nourishment containing ammonia.

ART. 21. *Comparative Return of the Sick of the Army serving in the Windward and Leeward Islands and Colonies from* 1803 *to* 1814 *inclusive.* By ROBERT JACKSON, M. D.

" THE treatment (says this veteran) of febrile diseases varied considerably within the period of the return alluded to; but I cannot, at present, give a full detail of it. At one time, and with one set of men, direct stimulation by brandy, wine, and opium, and nourishing diet, constituted the basis of the practice; cold affusion, according to the view of Dr. Currie, also had its day of fashion. It was succeeded by purgatives, as recommended by Dr. Hamilton; but the exhibition of mercury, (calomel internally and mercurial ointment by friction) in expectation of exciting salivation, which was considered as a cure, has been throughout the most prevailing of the routines. Whatever practice may have been followed, a comparative view of the returns from 1803 to 1811 inclusive, shews, that the results are not very different; in so far, at least, as can be supposed to depend on treatment. From 1812 to 1814 inclusive, the view of practice, more particularly at Barbadoes, was directed by a different principle, viz. by arresting the diseased action forcibly; and, where that was effected, by stimulating strongly to a train of action analogous with that of health. The subtraction of blood was the principal of the means employed with a view to effect the arrest; and the purpose was not attained, in many cases, by a less quantity than six pounds at one bleeding. The affusion of cold water on the head and shoulders, emetics, blisters to various parts of the body, stimulating purgatives, with diaphoretics, and frictions of the skin with warm oil, were among the stimulating means, employed to excite the counteraction, and to maintain its course. It is worthy of remark, that no instance of dropsy occurred in the hospitals at Barbadoes among persons who had been treated as here described; that nine out of ten were returned to duty in perfect health, within the fourteenth day; but there were few instances of imperfect cure, or visceral congestion; and that full diet and large allowances of wine, as appears by the table annexed to the return, were not productive of benefit." P. 283.

The 22d Article contains a short account of an Hepatic Abscess, which was opened by Mr. Astley Cooper, when a considerable quantity of fœtid matter was discharged. The patient sunk, however, a few days afterwards. The liver was found adherent to the peritonæum around the puncture (a little below the ensiform cartilage), but a large abscess in its substance. A central stricture of the stomach was also found. During the continuance of the disease, the patient suffered great pain at the stomach, which could not retain any solid food.

The work concludes with a Biographical Notice of Dr. James Johnstone, by J. C. Lettsom, M. D. and to the work itself we must refer for the particulars.

The contributors to this volume are highly respectable; the contributions interesting, and various; the information practical, and consequently useful: we therefore recommend its perusal.

